# Operationalizing anthropological theory: four techniques to simplify networks of co-occurring ethnographic codes

**DOI:** 10.1007/s41109-023-00547-6

**Published:** 2023-05-05

**Authors:** Alberto Cottica, Veronica Davidov, Magdalena Góralska, Jan Kubik, Guy Melançon, Richard Mole, Bruno Pinaud, Wojciech Szymański

**Affiliations:** 1 Edgeryders, Tallinn, Estonia; 2 Rutgers University, Newark, USA; 3 University College of London, London, UK; 4 Monmouth University, West Long Branch, USA; 5 CNRS, Bordeaux INP, LaBRI, UMR 5800, University of Bordeaux, Talence, France; 6 University of Warsaw, Warsaw, Poland

**Keywords:** Sociology, Anthropology, Ethnography, Networks, Visual analytics

## Abstract

The use of data and algorithms in the social sciences allows for exciting progress, but also poses epistemological challenges. Operations that appear innocent and purely technical may profoundly influence final results. Researchers working with data can make their process less arbitrary and more accountable by making theoretically grounded methodological choices. We apply this approach to the problem of simplifying networks representing ethnographic corpora, in the interest of visual interpretation. Network nodes represent ethnographic codes, and their edges the co-occurrence of codes in a corpus. We introduce and discuss four techniques to simplify such networks and facilitate visual analysis. We show how the mathematical characteristics of each one are aligned with an identifiable approach in sociology or anthropology: structuralism and post-structuralism; identifying the central concepts in a discourse; and discovering hegemonic and counter-hegemonic clusters of meaning. We then provide an example of how the four techniques complement each other in ethnographic analysis.

## Introduction

Since their inception, the social sciences have been split between qualitative and quantitative approaches. One of their most challenging undertakings has been to develop multi-method approaches that combine qualitative and quantitative techniques in ways that make them superior to both purely qualitative and purely quantitative methods. In this paper, we reflect on how to achieve such a combination in the practice of ethnography, a type of research that studies cultural phenomena from the point of view of the group that is being studied.

In ethnography, qualitative approaches are employed at the stage of data collection—via in-depth interviews—and at the stage of analysis, when the ethnographically established contextual knowledge is employed in an iterative interpretation of the collected material in order to reveal repeated, and thus in some sense “deeper”, patterns of thought. This interpretive work typically takes the form of an activity known as *coding*, where ethnographers associate keywords or keyphrases, known as *codes* to fragments of the text they analyse. We analyse the pattern of connectivity across codes, and render them in compelling visualisations. In these visualisations, an ethnographic collection of data, known as a *corpus* is represented as a network (Cottica et al. 2020), whose nodes correspond to ethnographic codes; the edges connecting them represent the co-occurrence of codes in the same part of the corpus. We call this network a *codes co-occurrence network* (henceforth CCN). In the paper, we interchangeably use the terms network or graph to denote the same entity formed of nodes (codes) and edges.

A problem that commonly arises is that the resulting networks are too large and dense for human analysts to process visually. Network science has come up with several (quantitative) techniques to simplify networks, based on identifying and discarding the least important edges in a network. It is relatively easy to apply them to this type of graph. What is harder is to justify the choice of one or the other of these techniques, and of the values assigned to the parameters that they usually require. These choices are all the more important in the current context of growing doubts about the epistemological status of data processing (Beaulieu and Leonelli 2021). Our objective is to contribute to the rigour and transparency of the methodological choices of researchers when dealing with large ethnographic corpora.

This paper builds upon, and extends, previous work in which we propose criteria for choosing a technique to simplify a CCN, and evaluate four candidate techniques against those criteria (Cottica et al. 2022). There, we highlight the affinity of each of the four techniques with a prominent method of analysis associated in turn with an identifiable school of thought in sociology or anthropology, using data from a study on Eastern European populism. Those results are summarised here in Sect. An application. The contribution of the present paper consists in (1) a complete consideration of how and why each technique for network simplification supports prominent methods in sociology and anthropology, and (2) a quantitative analysis of how the techniques in question perform on datasets constructed from three different ethnographic studies.

We proceed as follows. After discussing work related to our own (Sect. Related work), we introduce the codes co-occurrence network, which is the network to be simplified (Sect. The codes co-occurrence network). Next, we lay out criteria for choosing a technique to simplify a CCN for qualitative analysis, and introduce four such techniques (Sect. Techniques for network simplification). We then propose a mapping of our simplification techniques onto methods of analysis widely used in sociology or anthropology, and discuss the extent to which they produce similar results (Sect. Discussion) after examining how simplification techniques compare (Sect. Comparing simplification techniques). Finally, we proceed to apply them to our data, to show how the choice of a simplification technique sheds light on a specific facet of the studied phenomena (Sect. An application) and offer some concluding remarks (Sect. Conclusions and future research).

## Related work

The turn towards big data, fuelled by improvements in computing power, has led to renewed faith in the ability of quantitative work to provide more generalisable and yet valid knowledge (that is, knowledge that preserves some of the richness of case-derived insights) than that obtainable by qualitative studies or quantitative projects relying on smaller numbers of cases (Beaulieu and Leonelli 2021).

This has led to undeniable progress. At the same time, however, it has highlighted a need for methodological robustness. As scientific work based on large datasets becomes methodologically innovative, more steps are needed to move from raw data^1^ to final result. As a consequence, the methods themselves may become harder to check against the insights derived from intimate familiarity with specific cases. In combination with “publish or perish” and with the premium placed by journals on strong results [known as “publication bias” (Turner 2013)], this has led to various epistemological crises. The replication crisis in social psychology is the most famous of them (Maxwell et al. 2015). But there are others: for example, it is claimed that half of the total expenditure on preclinical research in the US goes towards non-replicable studies (Freedman et al. 2015). Other tendencies that worry quantitative scientists, and data scientists in particular, are: the persistence of citations of retracted papers (Economist 2021); the use of biased, bad-quality data in machine learning papers (Roberts et al. 2021); and the uncritical acceptance of raw data as representative of base reality, when in practice data are constructed (Beaulieu and Leonelli 2021; Leonelli 2019). All this leads to researchers obtaining divergent results depending on ostensibly innocent choices about data cleanup prior to analysis (Decuyper et al. 2016). Even controlled experiments with different researchers working with the same datasets on the same research questions have led to spectacularly divergent results, for reasons that are not yet entirely clear (Silberzahn et al. 2018; Breznau et al. 2021).

Qualitative sociological and anthropological research is not expected to be replicable; rather, its claim to generating reliable knowledge comes from the rigour and accountability of the methods applied systematically and self-consciously to a specific case or a small range of cases in well specified spatial and temporal contexts. Therefore, careful, transparent choices about one’s method are necessary at every step of the way, even more so when research applies mixed methods (Beaulieu and Leonelli 2021). This paper is meant as a contribution to applying this logic of transparency to the decision about how to simplify semantic networks that express qualitative data.

The literature on semantic networks originates in computer science (Sowa 1983, 2000; Woods 1975; Shapiro 1977); its main idea is to use mathematical objects—graphs—to support human reasoning. Branching out from this tradition, we focus on the idea of network simplification. The latter is useful because it makes the networks in question more amenable to visual analysis, and helps researchers to appreciate, and interpret, the patterns of connectivity in their data. In doing so, we factor in previous work on the cognitive limits of humans to correctly infer the topological characteristics of a network from visual inspection (Ghoniem et al. 2005; Melançon 2006; Munzner 2014; Soni et al. 2018). Such work confirms that large and dense networks are hard to process visually, and supports the case for network simplification.

Many avenues have been explored in order to tackle the analysis and visualisation of larger graphs. A widely studied approach consists in coarsening a graph to infer another graph with a smaller number of nodes and edges, an approach popularised by the seminal work of Newman and Girvan (2004), Fortunato (2010). The nodes of the resulting graph thus correspond to clusters, or communities, of nodes in the original graph. Although improving the readability of the data, the interpretation of the output of these algorithms can be challenging. Indeed, on top of being non-deterministic, non-expert users can find it difficult to give a clear meaning to these groups in terms of the underlying data.

Computing the backbone of a graph addresses the problem from another angle by discarding nodes and edges keeping those that form the skeleton of the graph. Because the simplified graph consists of nodes and edges from the original graph they remain interpretable in the original context. Most approaches promote the use of a metric, often designed with specific properties, to which a threshold is then applied either locally or globally (Herman et al. 1999; Nick et al. 2013; Coscia and Neffke 2017). It is this simplification approach we focus on in this paper, relying on four popular metrics.

It is important to maintain full awareness of the implications of applying each technique. In this sense, this work is inscribed in the tradition of scholars who aim to apply systematic visualisation techniques, while still retaining sensitivity to informants’ contextual, interactional, and socioculturally specific understandings of concepts (Dressler et al. 2005; Hannerz 1992; Strathern 1996; Burrell 2009). In doing so, we are aware of the potential accountability issues—and even crises—that could come with the adoption of mixed methods. To prevent them, we fashion our mathematical techniques so that they do not violate the specific requirements of knowledge creation in ethnography.

## The codes co-occurrence network

### Construction and interpretation

Consider an ethnographic corpus. In what follows, we call any text data encoding the point of view of one informant (interview transcript, field notes, post on an online forum and so on) a *contribution*. Contributions are then coded by one or more ethnographers. Coding consists of associating snippets of the contribution’s text to keywords or keyphrases, called *codes*. The set of all codes in a study constitutes an ontology of the key concepts emerging from the community being observed and pertinent to that study’s research questions^2^.

We can think of such an annotated corpus as a two-mode network. Nodes are of two types, contributions and codes; the same code can be used to annotate multiple contributions, and to annotate the same contribution multiple times. By annotating a contribution with a code, the ethnographer creates an edge between the nodes representing, respectively, the contribution and the code, resulting in a multiple edge graph.

**Fig. 1:**
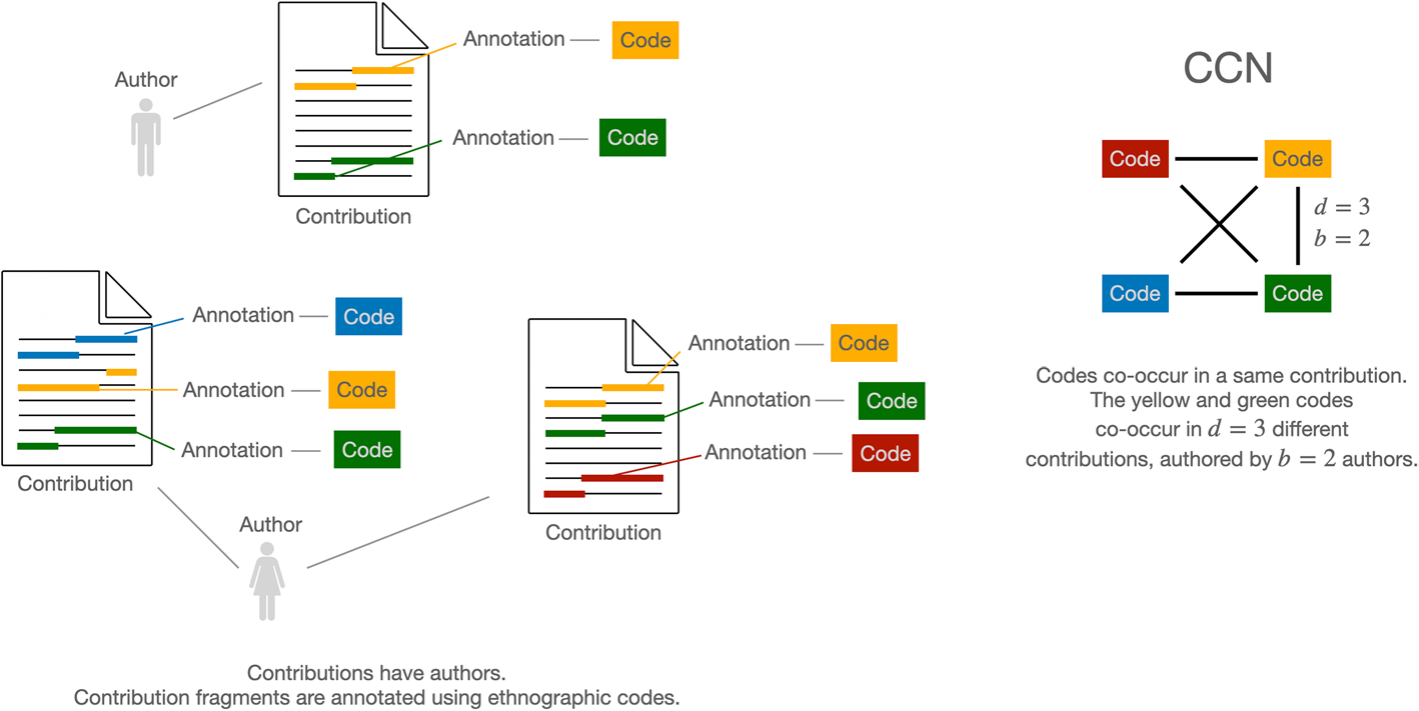
Inducing co-occurrence edges between ethnographic codes

From the two-mode network described above, we induce, by projection, the one-mode *codes co-occurrence network* (henceforth CCN). This is a network where each node represents an ethnographic code. An edge is induced between any two codes for every contribution that is annotated with both those codes (Fig. 1). This network is undirected ($A \rightarrow B \equiv B \rightarrow A$). There can be more than one edge between each pair of nodes.

This representation is both intuitive and useful. It is intuitive because it has a clear-cut interpretation. We interpret co-occurrence as association. If two codes co-occur, it means that one informant has made references to the concepts or entities described by the codes in the same contribution, seen as a unit. Hence, we assume, both concepts belong to this person’s culture-generated mental map. The whole set of such co-occurrences in the collected corpus of data is represented by a CCN.

The downside of CCNs is that they tend to be resistant to visual analysis. This is because they are large and dense. They are large because a large study is likely to use one to two thousand codes. They are dense as a result of the interaction of two processes. The first one is ethnographic coding. A rich contribution might be annotated 10 or 20 times, with as many codes associated to it. The second one is the projection from the 2-mode codes-to-contribution network to the 1-mode co-occurrence network. Recall that, in the latter, two codes are connected with an edge whenever they occur on annotations that annotate the same contribution. So, by construction, each contribution gives rise to a complete network (also called a clique) of all the codes associated to it, each of which is connected to all the others. Large, dense networks are known to be difficult to interpret by the human eye (Ghoniem et al. 2005; Melançon 2006; Munzner 2014). This is unfortunate, because we have found visual analysis of CCNs to be useful in generating insight, as well as new research questions (Keim et al. 2008; Kohlhammer et al. 2011). This is especially (but not only) true for ethnographers with little mathematical training (Cottica et al. 2020).

### Data and pre-processing

We use as data the annotated corpora from three ethnographic studies. One (OPENCARE) concerns community-produced health and social care services (Cottica and Melançon 2016); the second (NGI Forward, henceforth NGI), a policy-oriented discussion on the future of the Internet (Cottica and Hassoun 2021a); the third (POPREBEL), the lived experience of Eastern European populist politics (Cottica and Hassoun 2021b). Though very different in scope, the communities being studied, and the languages of the contributions, the corpora are roughly similar in size, each with about 4000 contributions by 300–400 informants. Their coding intensity is also roughly similar, with about 6,000 annotations and 1000–1500 codes each (Table 1).

**Table 1:** The datasets used: some descriptive statistics

	OPENCARE	NGI	POPREBEL
Informants	276	331	366
Contributions	3737	4068	3686
Annotations	5731	5871	6660
Codes	1391	1109	1605

To compare the results of the four techniques, we use the scripting capabilities of Tulip (Auber et al. 2017; https://tulip.labri.fr) for all graph processing and proceed as follows: first, from each dataset we induce the relative CCN. The resulting CCNs are too large and dense for visual analysis (Table 2). Second, we apply to each CCN different techniques for network simplification. The techniques and the rationale for choosing them are the subject of the next section. All techniques considered apply a simplification algorithm, the effects of which can be calibrated using one or two tuning parameters.

**Table 2:** Numbers of elements in the CCNs induced from the corpora of the three studies

	OPENCARE	NGI	POPREBEL
Nodes	1391	1109	1605
Edges	25,720	149,971	106,369

For each corpus and each technique, we then observe how varying the value of the tuning parameter influences the resulting simplified network. We attempt to find interpretations for choosing specific values of the tuning parameter.

Next, for each corpus and each technique we compute the maximal interpretable simplified network. By this, we mean the largest possible network that is still amenable to visual analysis, based on the relevant literature on network visualisation (Ghoniem et al. 2005; Melançon 2006; Munzner 2014).

Finally, for each corpus we assess the extent to which different simplification techniques select the same codes. We do this by computing a similarity statistics between the maximal interpretable simplified networks that are obtained from applying the different techniques.

## Techniques for network simplification

Any network simplification entails a loss of information, and has to be regarded as a necessary evil. Simplification methods should always be theoretically founded, and applied as needed, and with caution. We propose four simplifications techniques, and argue that each one maps to a distinct theoretical tradition in the social sciences, particularly anthropology.

Following King et al. (1994), we propose that a good simplification technique should:

1. Usefully support ethnographic inference, understood as a simplifying interpretation of the emerging intersubjective picture of the world. The main contribution of network simplification to ethnographic inference is that it makes the CCN small and sparse enough to be processed visually (Melançon 2006; Ghoniem et al. 2005; Munzner 2014). A substantial part of the human brain’s capacity is allocated to processing images, so it makes sense to invest in good visualisations. A well-established literature—and techniques such as layout algorithms—help us define what a “good” network visualisation is Herman et al. (2000).
2. Reinforce reproducibility and transparency. Reproducibility means that applying the same technique to the same dataset will always produce the same interpretive result (even if the technique has a stochastic component). Transparency means that how the technique operates is clear to the researcher, who can therefore assess which technique best suits her purpose, and explain that assessment to her peers.
3. Not foreclose the possibility of updating via abductive reasoning. Algorithms alone do not decide how parameters should be set to get optimal readability. Readability of a dataset representing an ethnographic corpus depends on the research question that the researcher brings to those data. By implication, the values of the parameters must be co-determined by the ethnographers, who possess rich empirical and theoretical knowledge of relevant contexts.^3^
4. Combine harmoniously with other steps of the data processing cycle, such as coding and network construction. This means making sure that the interpretations of the data and their network representation are consistent across the whole cycle.

With that in mind, we turn introducing our candidate techniques. We claim that all of them satisfy more or less equally Conditions 3 (parameters are set by the researchers), 4 (in ethnographic research, representing a corpus as a network of co-occurring codes is an established technique, and ethnographers find visual analysis of such networks fairly intuitive and compelling), and the reproducibility condition in 2 (the only stochastic components come into play in layout algorithms, and they produce visually equivalent layouts). However, they differ on how useful the visualisations they produce are (Condition 1: support for inference), and on how intuitive the method of building them is to ethnographers (Condition 2: transparency). Most of the discussion below therefore focuses on these two dimensions of usefulness and transparency.

### A first common step: merging multiple edges

Observe that the original graph we consider has multiple edges, that is nodes (codes) can be connected multiple times by distinct edges. Indeed, codes may co-occur in multiple contributions; they may even co-occur multiple times in a same contribution.

All four techniques require that we first merge multiple edges so that codes can only be connected through single edges. At the same time, the edges of the resulting graph are equipped with a weight function $\omega : E \rightarrow \mathbb {N}$. As we shall see, all four techniques use different weighting schemes.

### Association depth

As we already have underlined, pair of codes may co-occur multiple times. Hence, we may define a weight function $d: E \rightarrow \mathbb {N}$ where *d*(*e*) corresponds to the number of times a pair of codes co-occur in the original graph.

The value *d*(*e*) has an intuitive interpretation in the context of ethnographic research. Consider an edge $e = code1 \leftrightarrow code2$: it counts the number of times *code*1 and *code*2 co-occur.^4^ Since we interpret co-occurrence as association, it makes sense to interpret *d*(*e*) as the *depth of the association* encoded in *e*. This gives us a basis for ranking edges according to their value *d*(*e*). The higher the value *d*(*e*) of an edge, the more important that edge.

There is also a straightforward interpretation of the special case $d(e) = 1$. It means the association between +code1+ and +code2+ occurs only once in the corpus. It might be profoundly insightful, but it did not echo in the rest of the corpus. In a sense, it could represent the discursive isolate, an analogue of a statistical anomaly, an outlier. Dropping all edges *e* for which $d(e) = 1$ simplifies the network at what seems to be an acceptable cost.

Generalising, we can drop all edges for which $d(e) \le d^*$. As the value of $d^*$ increases, so does the degree to which the simplified network encodes high-depth associations between codes. Choosing an appropriate threshold $d^*$ below which to drop edges means managing a trade-off. The higher the threshold, the greater the information loss. At the same time, though, the higher the threshold, the greater the legibility of the simplified network, and the clearer the picture of the basic structure of discourse in a given community, within which our respondents create meaning and make sense of the world around them.

**Fig. 2:**
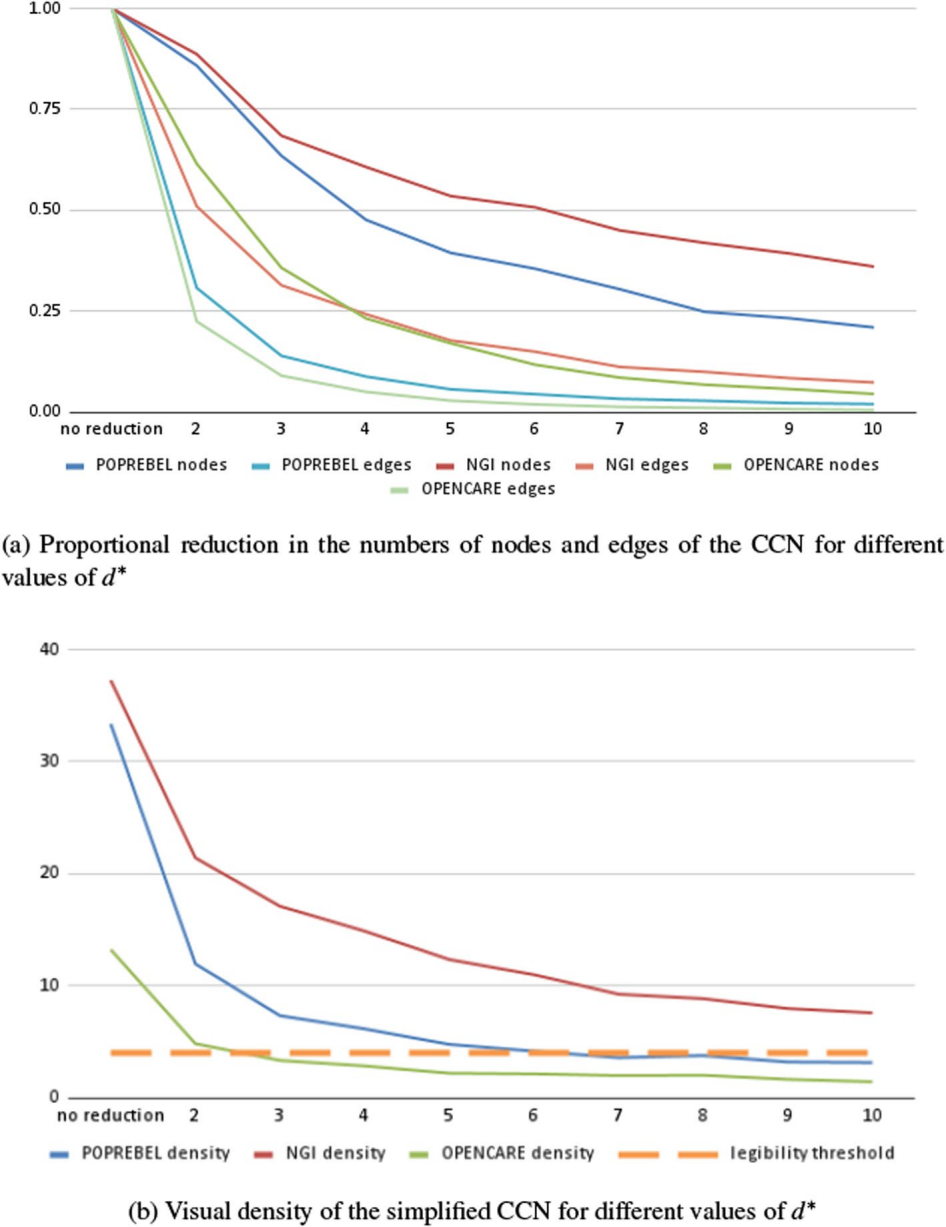
Simplifying codes co-occurrences networks, according to association depth, in three ethnographic studies

Figure 2a shows how the number of nodes and edges in the simplified co-occurrences networks of three semantic social network analysis studies decreases as we increase the value of $d^*$. Edges where $d(e) < d^*$ are simply discarded. Nodes whose incident edges were thus discarded, are discarded as well. Before simplification, the weighted networks for our three datasets have 1000 to 1500 nodes and 18,000 to 55,000 edges each. As $d^*$ increases, these numbers decrease rapidly.

Just setting $d^{*} = 2$ - which means only discarding one-off edges - leads, in our datasets, to a 50–75% decrease in the number of edges. A threshold of $d^{*} = 10$ leads to a decrease of about two orders of magnitude in the number of edges.

Figure 2b shows the decrease in network density (number of edges divided by the number of nodes) as the technique is applied with increasing values of association depth $d^*$. Un-simplified networks are very dense, with 15–40 edges per node. Discarding edges with $d(e) = 1$ reduces density by about half, but in two out of our three datasets densities remain well above the value of 4 edges per node, sometimes quoted as the one that makes for comfortable visual processing (Melançon 2006; Munzner 2014).

### Association breadth

A second weight function $b: E \rightarrow \mathbb {N}$ may be defined, where *b* is the number of informants who have authored the contributions underpinning those edges.

Recall that each edge *e* in the unweighted CCN is induced by one, and only one, contribution in the corpus, which was coded with both *code*1 and *code*2. This contribution has only one author. Instead of counting contributions to the corpus, like interviews or forum posts, we are counting the related informants. This also has a straightforward interpretation for ethnographic analysis. The greater the value of $b(e: code1 \leftrightarrow code2)$, the more widespread the association between *code*1 and *code*2 is in the community that we are studying. We interpret it as association breadth.

There is a mathematical relation between association breadth *b* and association depth *d*, namely $\forall e \in E: b(e)\le d(e)$.

Like for association depth *d*, the case where association breadth $b(e) = 1$ has a straightforward interpretation. It means the association between +code1+ and +code2+ is endorsed by only one single informant. It might be profoundly insightful, but it did not occur to anyone else in the community. It could reflect an idiosyncrasy of that particular person. Dropping all edges $e: b(e) = 1$ simplifies the network at what seems to be an acceptable cost.

As we did for depth, we can drop all edges for which $b(e) \le b^*$. As the value of $b^*$ increases, so does the degree to which the simplified network encodes broadly shared associations between codes. And again, the higher the threshold, the greater the information loss, and the greater the legibility of the simplified network, but thus also the clearer the picture of the cultural or ideological homogeneity in a studied community of discourse.

**Fig. 3:**
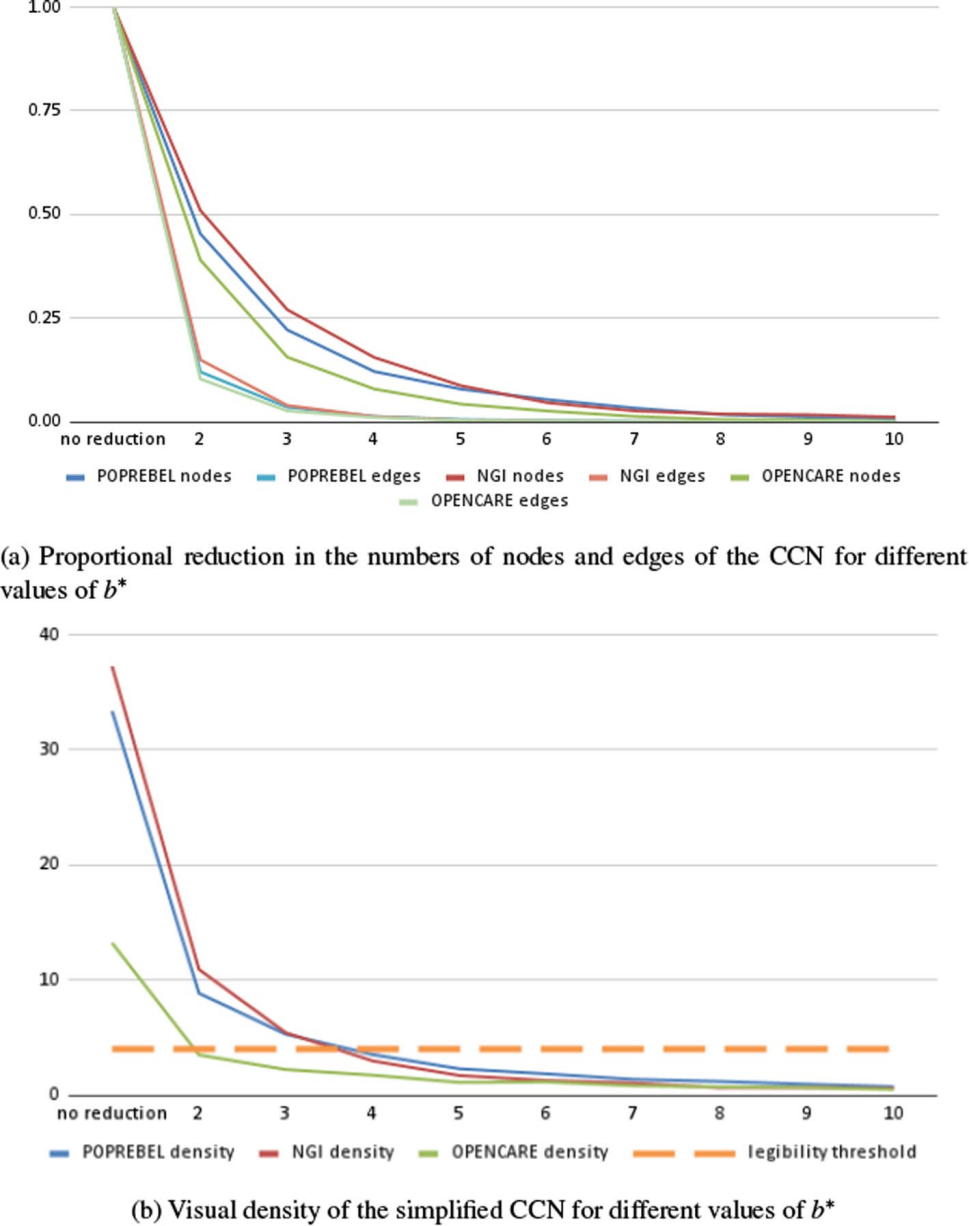
Simplifying codes co-occurrences networks, according to association breadth, in three ethnographic studies

Figure 3a shows how the number of nodes and edges in the simplified co-occurrences networks of three semantic social network analysis studies decreases as we increase the value of $b^{*}$. Setting $b^{*} = 2$ - which means only discarding “idiosyncratic” edges - leads to an 85–90% decrease in the number of edges. Setting $b^{*}$ to 4 leads to a decrease of about two orders of magnitude in the number of edges.

Figure 3b shows the decrease in network density (number of edges divided by the number of nodes) as the technique is applied with increasing values of association depth $b^*$. Discarding edges with $b(e) = 1$ reduces density by 70–75%, but again in two out of our three datasets they remain well above the value of 4 edges per node.

### Highest core values

An alternative way of identifying the most important edges in a CCN is to exploit the topology of the network. For example, a co-occurrence edge could be considered important if it connects two nodes that are both connected to a large number of other nodes. A community of such nodes can be identified by computing the CCN’s *k*-cores. *k*-cores are subgraphs that include nodes of degree at least *k*, where *k* is an integer. They are used to identify cohesive structures in graphs (Giatsidis et al. 2011). Random graphs have the property that a giant *k*-core appears in them when their edge density becomes high enough (Pittel et al. 1996).

After computing all the *k*-cores of a network, its nodes can be assigned a core value. A node’s core value is the highest value of *k* for which that node is part of a *k*-core.

To find the most important edges in the CCN, we remove all the codes whose core values *k* are smaller than 1, as well as their incident edges. If the graph thus simplified is still too large and dense, we increase the value of *k* to the next integer and repeat, until the simplified graph is amenable to visual analysis. Notice that this method uses weights on nodes rather than on edges as do the previously presented techniques. Co-occurrence edges are included in the simplified network only on the basis of the number of codes that the two co-occurring codes are connected to.

**Fig. 4:**
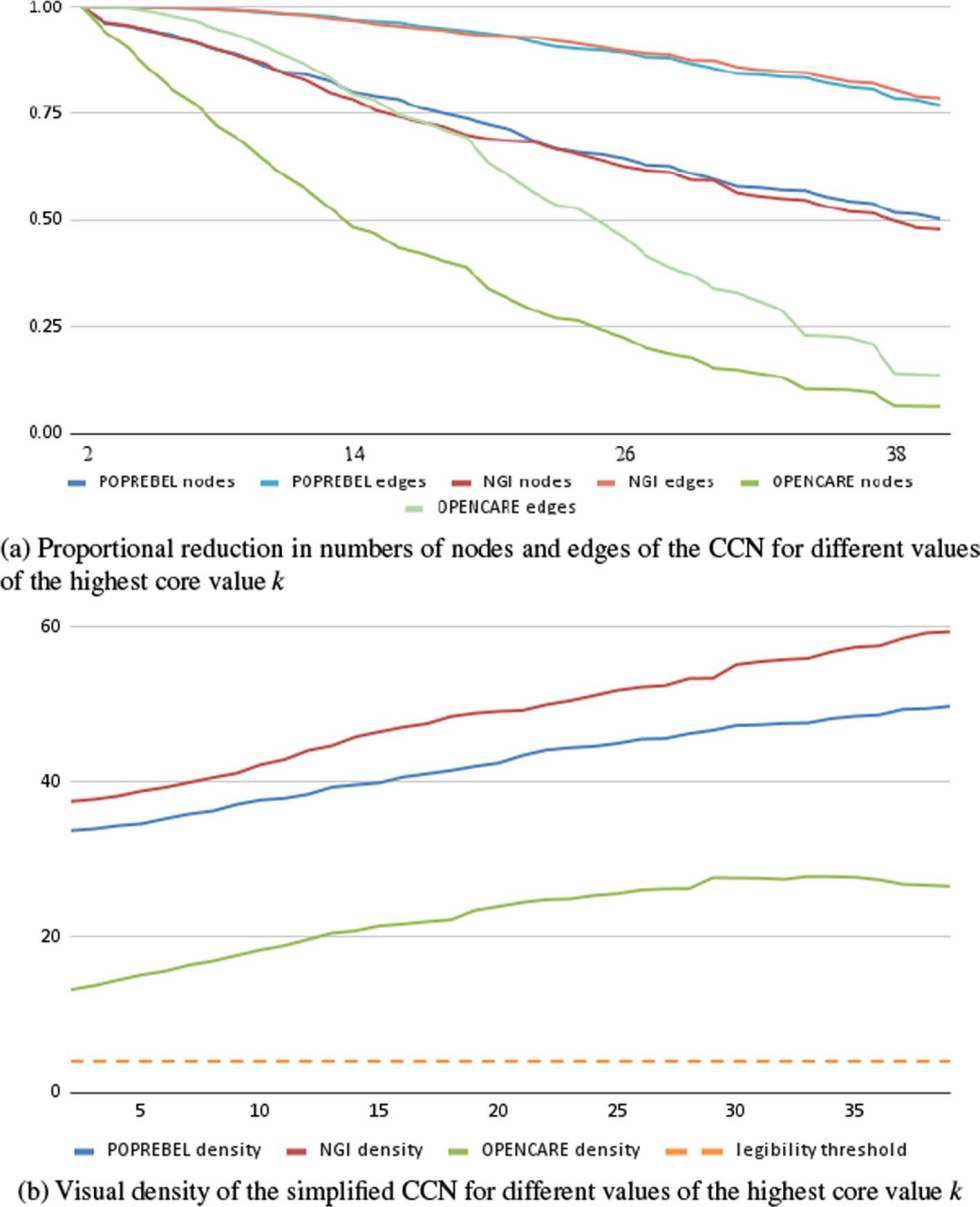
Simplifying codes co-occurrences networks, according to the core values of nodes, in three ethnographic studies

In contrast to the techniques presented above, this approach to network simplification is not very effective at low levels of the tuning parameter *k*. Only for high values of *k* does the CCN reach a substantial reduction in the number of nodes (under 100), and even then it maintains a very high number of edges (1000 to 10,000). As for edge density, it increases with *k*, staying well over the legibility threshold of 4. This is shown in Fig. 4.

Persistent high density and limited reduction in the number of edges are artefacts of the way in which *k*-core decomposition works. High-degree nodes are discarded last, so the highest-*k* core is composed only of nodes with many connections to one another.

In any affiliation network, like networks of co-authorship of academic papers or CCNs, the distribution of nodes’ core values is disproportionately influenced by the presence of very large “outlier” cliques. Some authors recommend dropping these cliques from the data manually (Giatsidis et al. 2011). In our case, a long and interesting contribution might be coded with as many as 50 codes. Each of those codes becomes connected to the other 49 in the CCN, driving their core values to at least 49, even if they do not appear anywhere else in the corpus. Moreover, in general, when the distribution of core values is fat-tailed, higher-*k* cores tend to be dominated by codes in the most heavily coded contributions, and so by the most vocal informants, who are able to deliver long and dense contributions. This is not necessarily what analysts want.

### Simmelian backbone extraction

Another way to exploit the network’s topology to identify its most important edges is to extract its Simmelian backbone. A network’s Simmelian backbone is the subset of its edges which display the highest values of a property called *redundancy* (Nick et al. 2013). An edge is redundant if it is part of multiple triangles. The idea is that, if two nodes have many common neighbours, the connection between the two is structural. This method applies best to weighted graphs. Both association depth and association breadth are natural measures of edge weight in CCNs. In what follows, we use the former weight function $d: E \rightarrow \mathbb {N}$.

This method requires we choose a value for a granularity parameter $\gamma$. We empirically set $\gamma$ to be equal to the average degree of the CCN induced from each dataset, rounded to the nearest integer. For each pair of codes $n_1, n_2$ in the network, redundancy is computed as the overlap between the $\gamma$ strongest-tied neighbours of $n_1$ and those of $n_2$. At this point, the network can be simplified based on the redundancy value of each edge. We start dropping the lowest-redundancy edges, then gradually increase the redundancy threshold to obtain smaller and smaller networks.

**Fig. 5:**
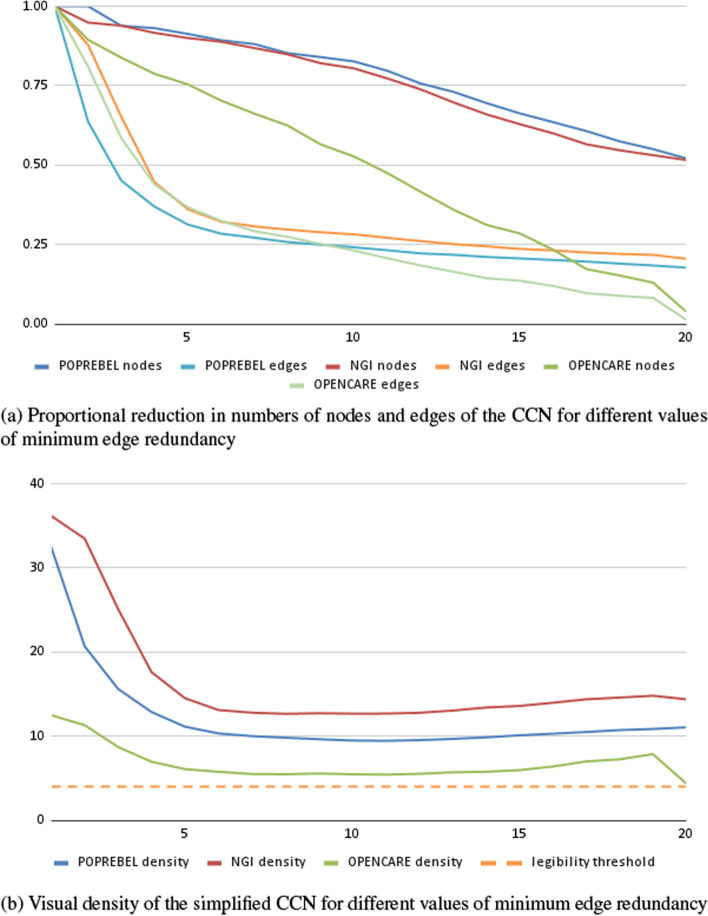
Simplifying codes co-occurrences networks, by the extraction of their Simmelian backbones, in three ethnographic studies

As we drop edges with higher and higher redundancy, the number of nodes decreases, but not very rapidly and with a more or less linear pattern for all datasets. The number of edges drops rapidly for low values of the minimum redundancy, but then decreases much more slowly when the network’s minimum redundancy rises above 5. Consequently, edge density sees a rapid drop in the early phases of the simplification, after which it becomes more or less constant. Throughout the simplification process, the density of all three datasets stays over the threshold value of 4 (Fig. 5).

Networks simplified with this method, while dense, appear more legible to human analysts than those simplified with the highest core values method. This is because, by construction, Simmelian backbone extraction tends to leave dense communities of nodes intact, while discarding edges that connect different communities. As a consequence, simplified networks are highly modular, and feature connected components breaking off the network’s main body. Networks simplified with this technique can be visually interpreted as small networks of communities of nodes, instead of large networks of individual nodes (see Fig. 7b). This appears to be semantically justified; the codes within each of the communities are in general semantically related. However, the same process tends to break the simplified network down into many densely connected components, which destroys structural information. So, with this technique, there is a trade-off between the reduction in the number of nodes and edges, on the one hand, and the preservation of a recognisable overall structure, on the other.

## Comparing simplification techniques

Simplifying any network implies ranking its edges in order of importance, so that the least important ones can be dropped for improved legibility. The simplification techniques we presented employ two different strategies to discover the CNN’s most important edges. Two of them—by association depth and by association breadth—rank edges according to the value that a chosen property, interpreted as edge weight, assumes for each individual edge. The other two techniques—by highest core values and by Simmelian backbone extraction—use the topology of the network to rank the importance of the edges (although the latter also employs a measure of edge weight to do so). Table 3 summarises, for each technique, the criterion to rank network edges, and its affinity with approaches commonly used in anthropology.

**Table 3:** Four techniques for edge ranking and their affinity to approaches in anthropology

Technique	Edges are important when...	In anthropology
Core values	...they connect two codes with high core values (Giatsidis et al. 2011)	“Central symbols in a culture”: Turner, Steward, symbolic anthropology
Simmelian backbone	...they are highly redundant (Nick et al. 2013)	“Culture as a field of competing forces”: Gramsci, Laitin, Comaroff
Association depth	...they encode a co-occurrence that occurs many times in the corpus	Structuralism and post-structuralism (Lévi–Strauss etc.)
Association breadth	...they encode a co-occurrence that occurs in the contributions of many informants	

**Fig. 6:**
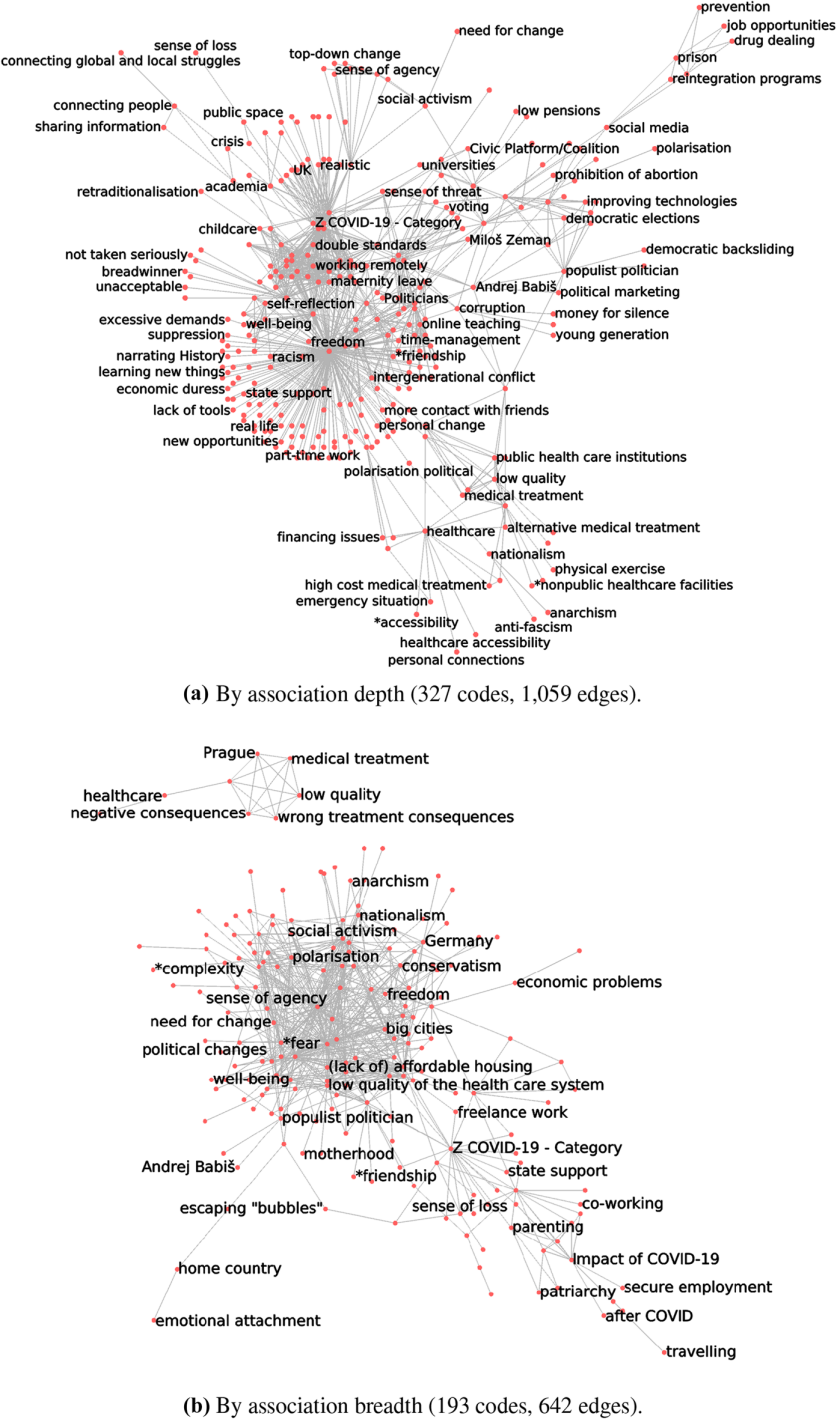
Reduced networks of the POPREBEL CCN (1/2, see Fig. 7)

**Fig. 7:**
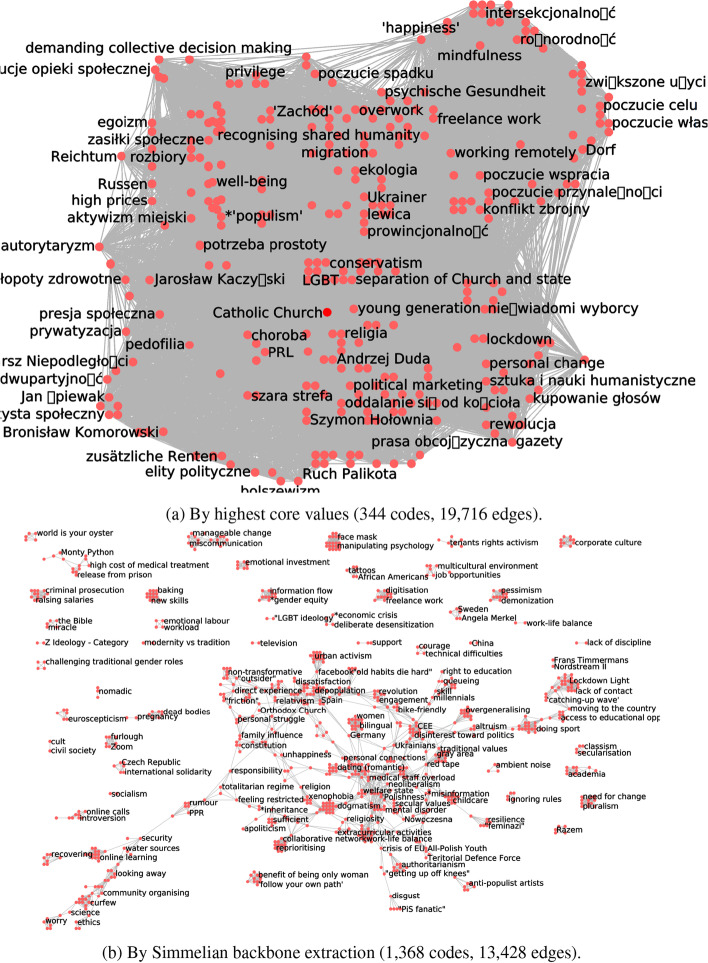
Simplified networks of the POPREBEL CCN (2/2)

In this section we compare the relative merits of the two strategies and four techniques, based on the criteria set at the beginning of this section. We discuss four aspects: interpretation of the simplification techniques themselves; harmonious integration with the pre- and post-simplification phases of the data processing cycle; quantitative effectiveness; and preservation of structural information in the simplified networks. This discussion is summarised in Table 4 and exemplified by Figs. 6 and 7.

**Table 4:** A comparison of four CCN simplification techniques against our chosen criteria

Criteria	Ass. depth	Ass. breadth	Core values	Simmelian backbone
Inference	Yes	Yes	Somewhat	Yes
Transparency	Yes	Yes	Somewhat	Somewhat
Abductive reasoning	Yes	Yes	No	Yes
Harmonious combination	Yes	Yes	No	somewhat

The reproducibility criterion is met by all techniques equally

The two simplification techniques based on edge weight are likely to be more intuitive to qualitative researchers without extensive training in network analysis. The measures of edge weight we adopted are grounded in the familiar practice of ethnographic coding; this results in straightforward definitions of what a strong edge is, and how simplified networks are obtained. By implication, these techniques effectively combine network simplification with the early (ethnographic coding and network induction) and late (analysis of the simplified network) phases of the data processing cycle. Ethnographic coding drives the entire cycle.

Conversely, interpreting network simplification based on the highest core values of nodes or on Simmelian backbone extraction requires a certain amount of topological thinking. While doing so is certainly possible to qualitative researchers, many of them are not specifically trained in it. In this sense, these two techniques are not as transparent as the former two. Like the previous two, these techniques combine with ethnographic coding upstream of simplification, but less so in the simplification phase itself, as edge weight is irrelevant to the highest core value of codes and only in part relevant to their “simmelianness”.

The two techniques based on edge weight allow for fine-tuning between high information loss and readability. They also allow us to produce small, low-density networks. Simplifying by highest core values does not allow such granularity, because the highest-*k* cores are still a substantial part of the networks before they undergo simplification; neither does it allow readability, because they are also dense, far above the threshold for visual analysis (Munzner 2014). The extraction of a Simmelian backbone has the same issues, but they are mitigated by two factors. First, the granularity parameter $\gamma$ can be increased to allow a more effective simplification. And second, the simplified networks are highly modular, and that allows for better readability for a given network size and the identification of clusters, if any, within it.

We now turn to how well structural information is preserved through network simplification. The first two methods yield simplified networks that preserve structural information, in the sense that their structure are not pre-determined by the method themselves: for example, the modularity (Newman and Girvan 2004) of the simplified networks varies across our different datasets. The third and fourth method use topological information for the simplification process itself, and they both predetermine the structure of the simplified network. Simplifying a CCN to the subnetwork formed by the codes with the highest core value invariably leads to a very dense network. The best a human analyst can do with it is ignore the edges altogether, and treat it as a list of important codes. Simplifying it to a Simmelian backbone invariably leads to highly modular simplified networks. As the value of the simplification parameter increases, communities of codes break off from the main body of the network and form entirely separate connected components; this highlights information about modularity, while concealing the overall pattern of connectivity in the corpus.

## Discussion

### Mapping network simplification techniques in sociology and anthropology

Deciding which network simplification technique is best suited to a particular research project will largely depend on the researcher’s ontological and epistemological beliefs, i.e., on assumptions about the nature of social reality and how this social reality can be known, as well as on the nature of the project itself, particularly the questions it asks.

Each of the four simplification techniques reveals a different set of attributes semantic networks have. It also turns out that the goals of each technique bear a family resemblance to the objectives of a prominent method of analysis associated in turn with an identifiable approach in sociology or anthropology.

Determining association depth is in its essence a method of uncovering the structure of a society or culture. Key works in anthropology (Lévi-Strauss 1958; Lévi-Strauss et al. 1962)—and in social theory (Althusser 1965; Poulantzas 1973) initiated a whole host of structuralist and post-structuralist approaches.

For post-structuralist sociologists and anthropologists, social relations can only be understood by analysing how they are constituted and organised through discourse. In other words, social hierarchies, norms and practices are legitimised by elevating specific concepts to a dominant position, enabling certain ideas to become widely accepted as the ‘Truth’. For example, the idea that ethnic nations are natural entities growing out of shared kinship ties (all academic evidence to the contrary) is used to legitimise political control by the core nation and the marginalisation of minority ethnicities. Moreover, discourse scholars work from the assumption that the meaning respondents attach to floating signifiers is relational within a discourse. Within a patriarchal discourse, the meaning attached to ‘woman’ is directly determined by the meaning attached to ‘man’, for instance. To understand the meaning of concepts, it is thus essential to understand their interrelationships; patriarchal discourse is identifiable by the concepts most often associates with the concepts of ‘woman’ and ‘man’. Focusing on association depth is thus a useful way of bringing into sharper focus the interrelationships between concepts that are most commonly used by our respondents. Such “core” interrelationships provide a picture of the basic structure of discourse in a given community, within which our respondents create meaning and make sense of the world around them.

For sociologists and anthropologists, the concept of association breadth is most closely associated with the study of networks, with Pierre Bourdieu’s work on social capital (Bourdieu and Wacquant 1992) and Jeremy Boissevain’s research on network analysis among the most influential (Boissevain and Mitchell 2018). Since it helps to identify an important attribute of networks not just among concepts but also actors who employ them, it seems to be particularly useful in reconstructing the structures of communities of discourse (Wuthnow 2009) or discursive fields (Snow et al. 2008). In short, this simplification method is designed to simultaneously capture information about connections between concepts and between people who employ them; it reveals networks emerging among the concepts used by the largest number of participants.

The technique based on core values of codes is designed to determine the centrality of certain concepts in a discourse. It facilitates, therefore, a more systematic determination which discursive elements constitute what is known in cultural anthropology as root paradigms, key metaphors, dominant schemas or central symbols of a given culture (Turner 1974; Aronoff and Kubik 2013).

Finally, the Simmelian backbone extraction can contribute to the discovery of hegemonic and counter-hegemonic clusters (subcultures) of meaning in an analysed body of discourse (Gramsci 1975; Laitin 1986). No society or culture is fully integrated and each is subjected to centripetal and centrifugal forces simultaneously. As a result, even in the most “homogenous” societies and cultures one can identify at least embryonic subcultures or—in another formulation—for every hegemony there is a budding or fully articulated counter-hegemony. The point is that a hegemony or counter-hegemony is usually built not on a single symbol or concept but on their interconnected cluster. This simplification technique helps to identify such clusters and assess with greater precision their shape and internal coherence.

### Do different techniques select the same codes and edges?

A priori, we expect different techniques to select into the simplified networks codes and co-occurrence edges that are different, but not completely different from technique to technique. Different techniques prioritise different edges, and, therefore, codes. At the same time, the key co-occurrences are likely to meet the criteria of every technique. In order to quantify the extent to which different techniques converge onto the same set of codes and edges, we proceed as follows.

First, we apply each of the four techniques to each of our three datasets. For each technique-dataset pair, we compute a maximal interpretable network (MIN). By this we mean the largest network that is still interpretable by a human analyst. We then take the four MINs of each dataset, and compare them pairwise by computing the Jaccard indices on their nodes and edges.

The main difficulty with the above is to define the MINs. While graph layout algorithms have focused on minimising edge crossing, symmetry, and other such layout properties, there is little research on how the visual representation of a graph influences the perception of quantitative properties of that graph (Soni et al. 2018). Some attempts have been made to correlate graph attributes (like density and order) with the ability of humans to correctly perceive basic graph properties like diameter or shortest paths (Ghoniem et al. 2005; Soni et al. 2018). We would instead like to use the CCN mostly to derive insights on the overall shape of the association patterns in a large corpus. In the absence of a systematic literature on the readability of graphs, we fall back on the result that graphs become difficult to interpret once their number of edges rises above four times the number of their nodes, confirmed by several authors (Ghoniem et al. 2005; Melançon 2006; Munzner 2014). The MIN, then, becomes the largest simplified graph for which $\frac{E}{N} < 4$, where *E* is the number of edges in the simplified network, and *N* the number of its nodes.

This criterion does indeed provide a MIN when applied to simplification based on *d*(*e*) and *b*(*e*) (see Sect. Techniques for network simplification). However, no amount of simplification based on highest core values and Simmelian backbone extraction yields a simplified network that satisfies it. For those techniques, we have to adopt other definitions of MIN.

For highest core values, we simply define the MIN as the size of the *k*-core with the largest value of *k* in the network before simplification. This MIN is much too dense to be visually interpreted, but it does provide the ethnographer with a list of codes, that constitute the highest-cohesion group of codes in the corpus.

For Simmelian backbone extraction, we exploit the property of Simmelian backbones to filter out edges connecting different communities of nodes, preserving those that connect different nodes in the same community. This greatly facilitates visual identification of communities of nodes (Nick et al. 2013). On the down side, as the value of the tuning parameter increases, this technique produces simplified networks that break down into several connected components. This process destroys information on how these communities connect to each other. The latter is clearly valuable to ethnographers, because it is a part of the structure of the discourse in a corpus. So, we define the MIN as the smallest Simmelian backbone of the original network in which a single component includes at least 80% of the codes.

**Table 5:** The maximal interpretable networks (MINs) obtained after applying our four techniques on the three datasets

Technique	OPENCARE		NGI		POPREBEL	
	Codes	Edges	Codes	Edges	Codes	Edges
Ass. depth	472	1575	214	837	476	1754
Ass. breadth	514	1799	173	551	193	642
Core value	1076	16,810	115	6555	172	9172
S. backbone	754	4764	570	12,067	879	13,180

Given these criteria, the number of nodes and edges in each MIN is summarised in Table 5. Tables 6 and 7 show the degree to which pairs of techniques choose the same codes and edges, as measured by their overlap coefficients. The overlap coefficient between two sets—in our case, the sets of codes or edges selected by two techniques—obtains by dividing the number of elements in both sets by the number of elements of the smallest of the two sets. Formally, if *A* indicates the number of elements in set *A*:

$$\begin{aligned} overlap(A,B) = \frac{A \cap B}{\min (A, B)} \end{aligned}$$

A value of 0 indicates no common element between the sets, whereas a value of 1 indicates that all the elements of the smaller set are also contained in the larger one. For codes, most overlap coefficients cluster around values greater than 0.5, which indicates that many codes are selected by many, potentially all, our techniques. This indicates that, while different techniques are best suited to exploring different research questions, all will include the core codes in an ethnographic corpus.

For edges, the comparison is less meaningful, because large differences in the number of edges in the different MINs reduce the values of the overlap coefficients. Nevertheless, similarities are visible between association depth and association breadth, and between core values and Simmelian backbone.

**Table 6:** Overlap coefficients between the sets of codes selected by each pair of simplification techniques

	Core value	Ass. breadth	S. backbone
Association depth			
OPENCARE	0.975	0.733	0.856
POPREBEL	0.564	0.777	0.811
NGI	0.174	0.659	0.949
Core value			
OPENCARE		0.951	1.000
POPREBEL		0.610	0.959
NGI		0.235	1.000
Association breadth			
OPENCARE			0.804
POPREBEL			0.933
NGI			0.960

**Table 7:** Overlap coefficients between the sets of edges selected by each pair of simplification techniques

	Core value	Ass. breadth	S. backbone
Association depth			
OPENCARE	0.992	0.583	0.331
POPREBEL	0.122	0.380	0.362
NGI	0.024	0.314	0.315
Core value			
OPENCARE		0.205	0.991
POPREBEL		0.199	0.146
NGI		0.407	0.601
Association breadth			
OPENCARE			0.331
POPREBEL			0.362
NGI			0.315

## An application

We use a subset of the POPREBEL corpus to show how each of the four simplification techniques can be seen as broadly corresponding to a paradigm in anthropology - a convergence that attests to the utility of such a synthesis. This application is not meant as a full methodological primer. Rather, it means to be a “proof of concept”, and shows the possibilities of synthesising quantitative and qualitative techniques in the service of ethnographic insight. More applications can be found in Davidov et al. (2022).

These data were gathered in the spring and summer of 2021 (Cottica et al. 2022). They consist of 17 semi-structured interviews with Polish-speaking Internet users, who used social media to seek and share information about health against the backdrop of the COVID-19 pandemic. Research participants were asked about their opinion on the current state of affairs in their respective countries, and their political choices over the years and at present. The interviews’ transcriptions (about 78,000 words) were then split into contributions, in the sense of Sect. The codes co-occurrence network: each question of the interviewer, and answer of the interviewee was considered as a contribution. In what follows, two codes are considered to co-occur if, and only if, they were both used in annotating the same contribution (as opposed to the same interview). Computed this way, the CCN from this corpus includes 1116 contributions, and 2152 annotations. The latter use 600 unique codes, connected by 16,370 co-occurrence edges.

We apply simplification techniques to the CCN in sequence, trying for different levels of the respective simplification parameters (*d*, *b*, *k*, *r*) in order to achieve a good combination of legibility (more edges discarded) and completeness (fewer edges discarded). In each simplified network, we focus on the ego network of one code in particular, Catholic Church. We selected this particular code in the expectation that the Catholic Church would be fairly central in any ethnographic study of populism in Poland. While there are, of course, many ways to approach network analysis, we were inspired to create an entry point by the conventions of kinship charts in anthropological research: a kinship chart must always have an “ego”—an individual through whom all kinship relations are traced, and to whom they all refer.

**Fig. 8:**
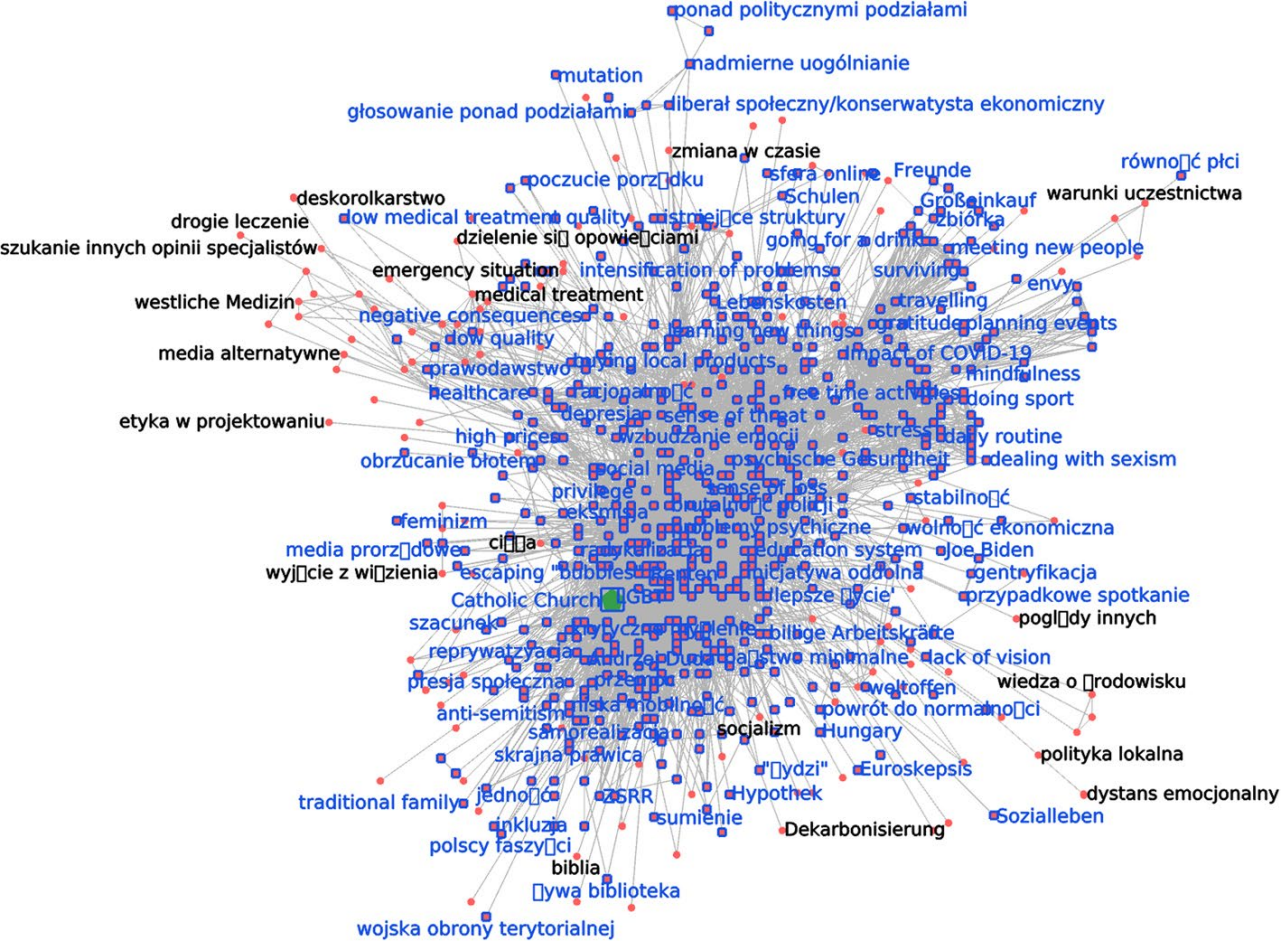
The full CCN. The 28-core is shown highlighted in blue. It contains Catholic Church (in green)

*Highest core values.* Anthropology as a discipline has a long history of trying to identify “core” dimensions of culture, both to better theorise how a given culture is constituted, and as a useful heuristic for ethnographic fieldwork (cf Boas’s outer and inner forces (Boas 1932), Kroeber’s reality and value culture (Kroeber 1950), Steward’s cultural core (Steward 1972). Victor Turner, considered to be a founding figure in symbolic anthropology, subscribed to a definition of symbol as “*a thing regarded by general consent as naturally typifying or representing or recalling something by possession of analogous qualities or by association in fact or thought*” (Turner 1975) and it is the recollection and association aspects that are of particular interest to us. While Turner did not seek to define a fixed core of concepts within a culture the way Steward, for example, did, he did write about symbols “variously known as ‘dominant,’ ‘core,’ ‘key,’ ‘master,’ ‘focal,’ ‘pivotal,’ or ‘central’ [that] constitute semantic systems in their own right [with a] complex and ramifying series of associations as modes of signification.” We envision the core value simplification revealing something akin to such a semantic system, one that we approach in the spirit of Turner’s notion of “positional meaning”—a level of symbolic meaning derived from analysing a symbol’s association to other symbols and cultural concepts. In our data, we see the highest core values simplification yielding an innermost nucleus of nodes (ethnographic codes) that recur most often in relation with each other. Catholic Church is close to the center of the symbols expressing this culture. Mathematically, it belongs to one of the innermost *k*-cores, ($k=28$, containing 82 codes, shown in Fig. 8), though not the absolute innermost. Only two *k*-cores exist in the graph where *k* is higher than 28 ($k=29$, $k=42$). The analysis supports the conclusion that the Catholic Church is one of the core symbols in this culture.

**Fig. 9:**
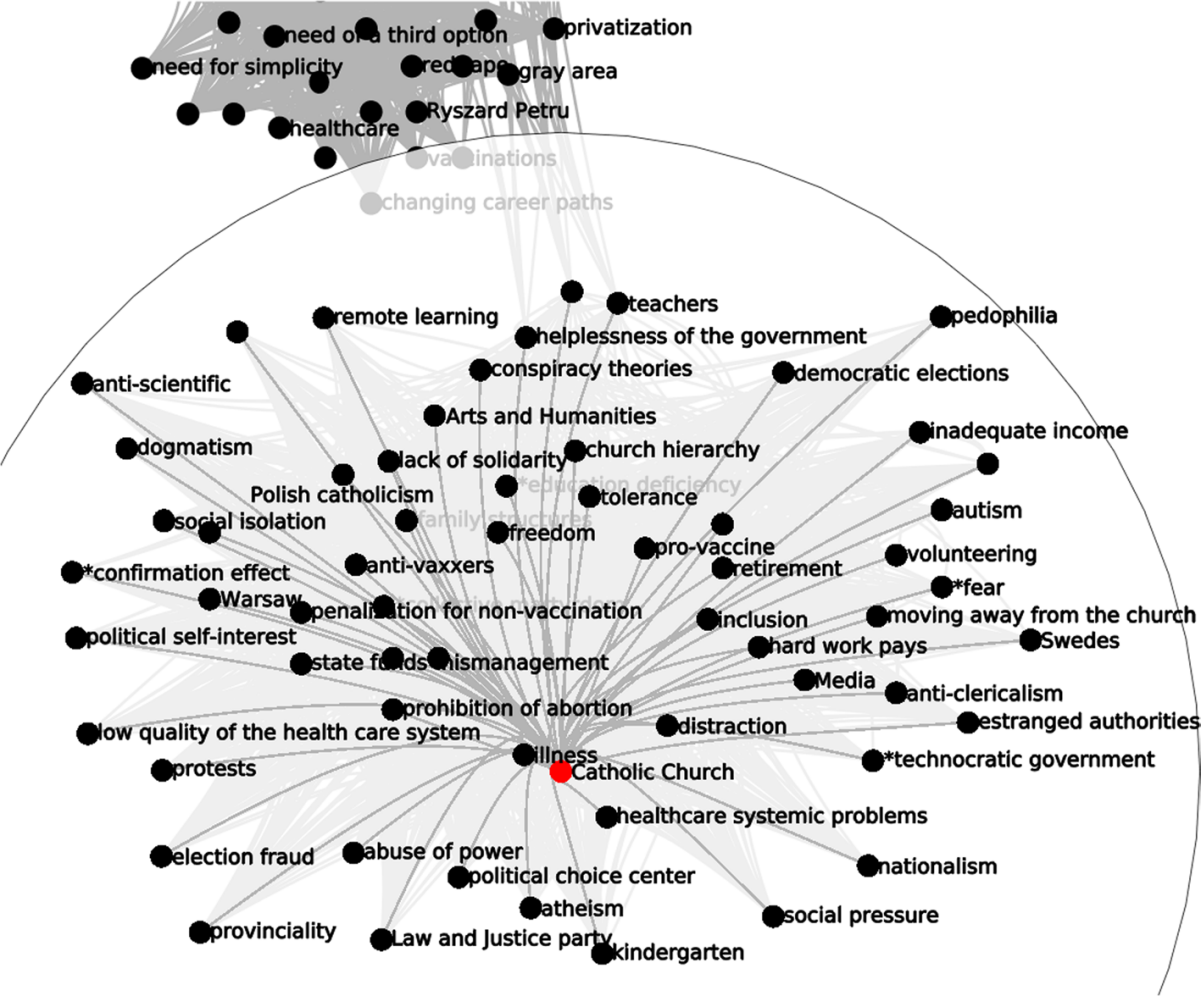
The ego network of Catholic Church, with only edges with edge redundancy $r>30$ shown

*Simmelian backbone.* Next, we explore the neighbourhood of Catholic Church through the lens of the Simmelian backbone simplification technique. While usually the Simmelian backbone technique is used to identify homophily and strong ties in a social network of actors (Nick et al. 2013), we are looking at the strong ties between concepts. In a way, when applied to concepts rather than human actors, this approach, in making visible strong associative links, literalises the notion of certain ideas being “in conversation” with each other. The visualisation reveals several such “conversations”—a dynamic which maps onto the anthropological notion of culture as a field of competing forces. As Comaroff and Comaroff write, “culture [is] the semantic space, the field of signs and practices, in which human beings construct and represent themselves and others, and hence their societies and histories ... culture always contains within it polyvalent, potentially contestable messages, images, and actions.” Comaroff and Comaroff (2019) This approach allows us to see, from a bird’s eye perspective, how various “signifiers-in-action” (ibid.) coalesce into identifiable subfields of signs or semantic subspaces. Catholic Church belongs to a community of codes that are political rather than spiritual- such as abuse of power, political self-interest, and nationalism (Fig. 9). The ethnographic interpretation we have here is that people have concerns pertaining to the Catholic Church both in the context of what they conceive as this institution’s excessive politicisation and more personal concerns, anxieties, and anomic tendencies. This can be used as a foundation to build on iteratively in future research on a range of subjects, including but not limited to political cultures, epistemology, various dimensions of trust and belief, and the position of the Catholic Church in the public space and the country’s culture.

**Fig. 10:**
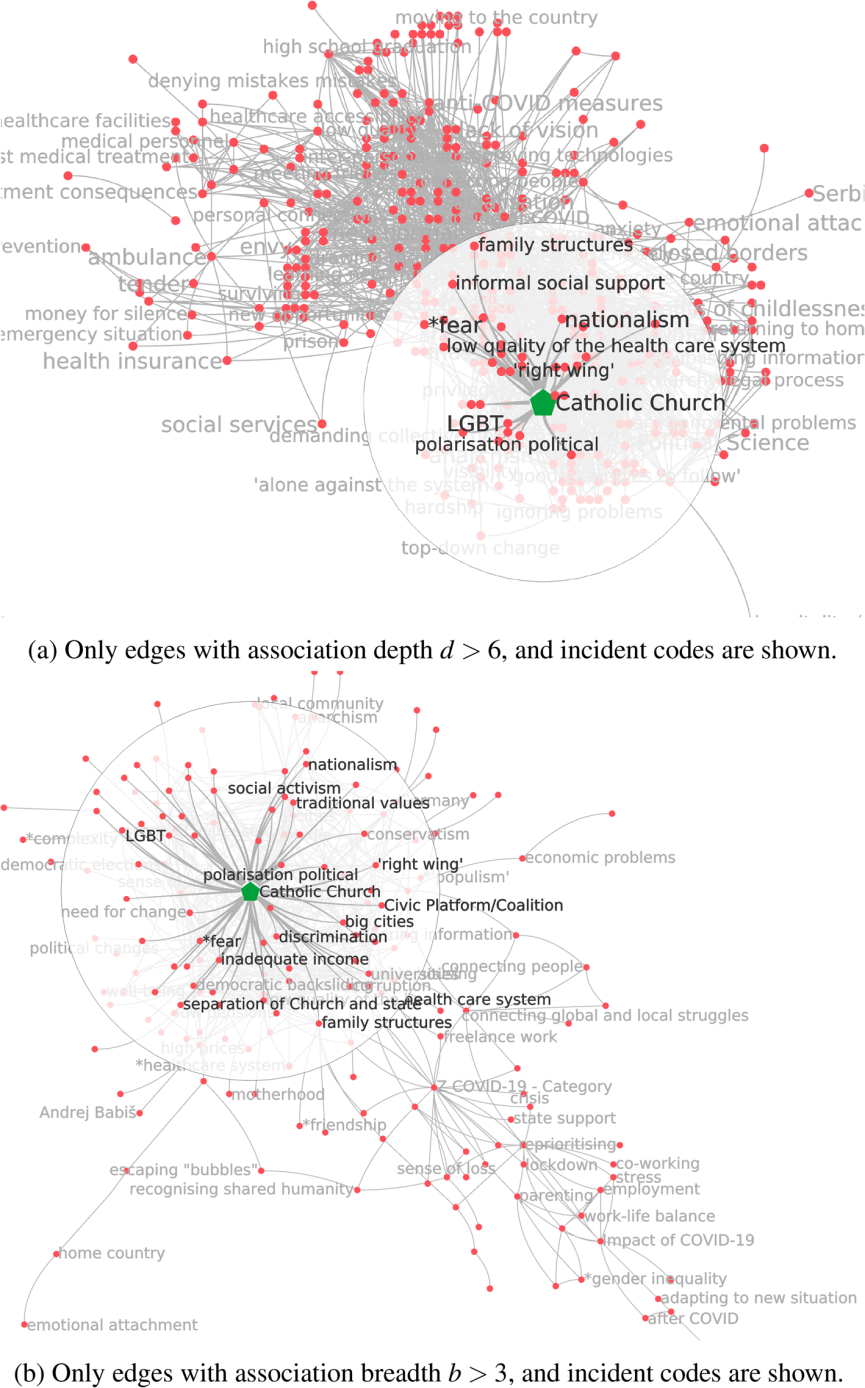
The ego network of Catholic Church, simplified by two different techniques

*Association depth and association breadth.* We now turn to the association depth and association breadth simplification techniques, which work in tandem to deepen our understanding of the underlying structures of discursive associations. The association depth visualisation shows us which associative links between concepts are the strongest—in other words, which codes emerge as being mentioned together most often. At the same time, association breadth helps evaluate the diffusion of these “deep” edges among informants. The association depth-simplified CCN, taken in isolation, might skew our perception of what ideas link up with each other, if, for instance, the depth of some edges was inflated by one or two informants repeatedly linking certain concepts with each other over and over again in their interviews. When the results produced through the depth and breadth simplifications align, we know that deep associations are not generated by a small number of interviews with people who frame a topic by linking it repetitively with a constant, limited set of other topics, but rather a broad agreement that emerges from the analysis of many interviews or conversations. We can see how this plays out with Catholic Church code (Fig. 10a): the three deepest associations are formed between it and the abuse of power, politicisation, and Polish catholicism codes. If we choose lower (but still significant, in the sense that the number of edges in the CCN is simplified by over 95%) levels of the tuning parameter *d*, codes like LGBT, discrimination and Law and Justice party appear.

The association breadth-simplified CCN shows that the broadest links to Catholic Church are very similar to the deepest ones. The very broadest three connect it to politicization, Polish catholicism, and discrimination, and tolerance. Edges to “political” codes like LGBT, inequality, abuse of power and abortion resist to simplifications by over 50% in the number of edges in the CCN (Fig. 10b). In our case, these two simplification methods yield closely aligned results. Both attest to the Catholic Church figuring as an institution associated with politics more so than with faith or spirituality among the informants. Even though there are some codes visible in the graph that may correlate to spirituality, the broadest associations still link the Catholic Church with political codes and the issue of abuse of power.

## Conclusions and future research

The use of mixed methods enables exciting progress in sociology and anthropology. However, when using these methods, researchers have to make decisions that, though they might look innocent, are liable to introduce biases and unduly influence their conclusions. Advisable countermeasures include raising one’s awareness of one’s own methodological decisions; documenting them meticulously, so as to facilitate peer review and peer critique; and grounding them in theory and in the context of the inquiry. The challenge is to find techniques for data processing which are mathematically effective as well as theoretically justified. Applying this approach to the problem of simplifying networks of codes induced from ethnographic corpora has meant exploring our candidate technique both from a mathematical and from an interpretive point of view.

Several relevant directions of inquiry have been left to future research. A conceptually simple one, though difficult to operationalise, is to improve the criteria for selecting a MIN: the extant literature considers a network “readable” when subjects in an experiment can correctly answer questions about its mathematical properties. For a sociologist or an anthropologist, however, interpretability is more about the ability of a network to convey information about the overall structure of the discourse in the underlying corpus. Another promising direction is to compare topological and semantic proximity of the codes in a CCN; that is, to assess the extent to which communities of codes identified by applying algorithms are constituted by codes with closely related meanings. Finally, the mapping of simplification techniques onto schools of thought in sociology and anthropology we propose needs to be further fleshed out and tested. Some of this future research might be a good match for lab experiments.

## Data Availability

The (pseudonymized) datasets analysed during the current study are available in the Zenodo repositories: Cottica and Melançon (2016), Cottica and Hassoun (2021a), Cottica and Hassoun (2021b), Cottica et al. (2022). The codes co-occurrence networks were built from those same data, simplified, and visualized using Tulip. Both the Tulip graph file and the Python code to run the different simplifications algorithms under Tulip are available at 10.5281/zenodo.7880825/. The Python script is designed to be used inside the Tulip Python IDE. It builds a graph hierarchy which is used to get the number of nodes and edges for each simplification step to eventually construct Figures 2 to 5.

## Footnotes

1 Though the concept itself of “raw data” is deemed problematic (Beaulieu and Leonelli 2021).

2 For a complete description of the data generation process, see Sect. The codes co-occurrence network of Cottica et al. (2020).

3 User-defined values for parameter come with additional risk of introducing biases into the analysis. However, full objectivity is neither possible nor pursued in the social sciences (Beaulieu and Leonelli 2021). Ethnographers are trained to deal with the risk of bias via a series of techniques (rigour, iteration, radical transparency on their own views and potential sources of biases via positionality statements (Silva et al. 2018)) that apply to network simplification in the same way that they apply to all other phases of research; design, interviews, coding and so on.

4 Two codes can also co-occur multiple times in the same contribution. This happens when the ethnographer, in coding the contribution, uses the codes more than once. Typically this happens when the same code is evoked in two different sentences contained in the same contribution.
